# Systematic Review and Meta-Analysis of American College of Radiology TI-RADS Inter-Reader Reliability for Risk Stratification of Thyroid Nodules

**DOI:** 10.3389/fonc.2022.840516

**Published:** 2022-05-13

**Authors:** Wei Li, Yuan Sun, Haibing Xu, Wenwen Shang, Anding Dong

**Affiliations:** ^1^ Department of Medical Imaging, Jiangsu Vocational College of Medicine, Yancheng, China; ^2^ Department of Burn and Plastic Surgery, Affiliate Huaihai Hospital of Xuzhou Medical University, Xuzhou, China

**Keywords:** thyroid nodule, ultrasonography, reproducibility of results, classification, meta-analysis

## Abstract

**Purpose:**

To investigate the inter-reader agreement of using the American College of Radiology (ACR) Thyroid Imaging Reporting and Data System (TI-RADS) for risk stratification of thyroid nodules.

**Methods:**

A literature search of Web of Science, PubMed, Cochrane Library, EMBASE, and Google Scholar was performed to identify eligible articles published from inception until October 31, 2021. We included studies reporting inter-reader agreement of different radiologists who applied ACR TI-RADS for the classification of thyroid nodules. Quality assessment of the included studies was performed with the Quality Assessment of Diagnostic Accuracy Studies-2 tool and Guidelines for Reporting Reliability and Agreement Studies. The summary estimates of the inter-reader agreement were pooled with the random-effects model, and multiple subgroup analyses and meta-regression were performed to investigate various clinical settings.

**Results:**

A total of 13 studies comprising 5,238 nodules were included in the current meta-analysis and systematic review. The pooled inter-reader agreement for overall ACR TI-RADS classification was moderate (*κ* = 0.51, 95% CI 0.42–0.59). Substantial heterogeneity was presented throughout the studies, and meta-regression analyses suggested that the malignant rate was the significant factor. Regarding the ultrasound (US) features, the best inter-reader agreement was composition (*κ* = 0.58, 95% CI 0.53–0.63), followed by shape (*κ* = 0.57, 95% CI 0.41–0.72), echogenicity (*κ* = 0.50, 95% CI 0.40–0.60), echogenic foci (*κ* = 0.44, 95% CI 0.36–0.53), and margin (*κ* = 0.34, 95% CI 0.24–0.44).

**Conclusions:**

The ACR TI-RADS demonstrated moderate inter-reader agreement between radiologists for the overall classification. However, the US feature of margin only showed fair inter-reader reliability among different observers.

## Introduction

Thyroid nodules are very common in the general population and affect approximately half the population older than 40 years (1–3). Even though most thyroid nodules are benign, 5%–15% are proved to be malignant (4, 5). In recent years, the detection rate of thyroid nodules is increasing with the widespread use of ultrasound (US), and the incidence of thyroid cancer has increased about 5 times in the past 5 decades (6). Currently, the US is accepted as the most effective modality for routine thyroid nodule detection and evaluation and also is used as a guide during fine-needle aspiration biopsy (FNAB) (7–9). However, the risk stratification of thyroid nodules is primarily dependent on radiologists’ personal experience and deficiency of reproducibility and agreement, which results in many overdiagnoses and unnecessary FNABs (10).

To address this problem, various guidelines and scoring systems were developed to standardize the risk stratification and management of thyroid nodules, including the American Thyroid Association (ATA) guideline (10, 11), Kwak Thyroid Imaging Reporting and Data System (TI-RADS) (12), American Association of Clinical Endocrinologists (AACE) guidelines (13), and European Union (EU) TI-RADS (14). In 2017, the American College of Radiology (ACR) released the ACR TI-RADS, in which a thyroid nodule is scored 1–3 points according to five US features (composition, echogenicity, shape, margin, and echogenic foci). Afterward, the calculated sum of these points is used to classify a nodule into one of five malignancy risk groups: benign (TR1, 0 points), minimally suspicious for malignancy (TR2, 2 points), mildly suspicious for malignancy (TR3, 3 points), moderately suspicious for malignancy (TR4, 4–6 points), or highly suspicious for malignancy (TR5, ≥7 points). Since the ACR TI-RADS was proposed, it has been one of the most reported guidelines in recent years (15, 16). In a recently published study, Liu et al. reported that the inter-observer agreement for ATA guidelines was 0.628 and for ACR TI-RADS was 0.748 (17). However, the inter-reader agreement of this scoring system has not been systematically evaluated. Therefore, in this study, we aimed to assess the inter-reader agreement of the ACR TI-RADS for the classification of thyroid nodules.

## Methods

For this meta-analysis and systematic review, a standardized review and data extraction protocol was used, performed in compliance with the Preferred Reporting Items for Systematic Reviews and Meta-Analyses (PRISMA) statement (18). The primary outcome was the inter-reader agreement using ACR TI-RADS for risk stratification of thyroid nodules.

### Search Strategy and Selection Criteria

A thorough literature search was performed of PubMed, EMBASE, Cochrane Library, Web of Science, and Google Scholar from inception until October 31, 2021, with no language restrictions applied. Original research articles reporting inter-reader agreement of ACR TI-RADS were identified, and the terms combined synonyms used for searching were as follows: ([ACR] OR [American College of Radiology]) AND ([TI-RADS] OR [TIRADS] OR [Thyroid Imaging Reporting and Data System]) AND ([thyroid nodule] or [nodule]). An additional search was performed by manually screening the bibliographies of the included articles and reviews. Studies identified by literature search were assessed by two independent reviewers (WL and YS, with 6 and 8 years of experience, respectively, in performing systematic reviews and meta-analyses) for potential inclusion, and disagreements were resolved by consensus *via* discussion with a third reviewer (AD).

### Inclusion and Exclusion Criteria

Studies that satisfy all of the following criteria were included: 1) studies that reported the inter-reader agreement of ACR TI-RADS for risk stratification of thyroid nodules; 2) studies that provided Cohen’s kappa (*κ*) values or intraclass correlation coefficients (ICCs) and their 95% CIs, or other measurements for assessment of inter-reader agreement; and 3) cytology results from US-guided FNAB or surgical resection pathology results as the reference standard. Studies that met any of the following criteria were excluded: 1) with a small sample size that involved less than 20 participants; 2) studies using other scoring systems rather than the ACR TI-RADS; 3) studies that did not provide detailed data to evaluate the inter-reader agreement; and 4) not original articles such as reviews, letters, guidelines, conference abstracts, or editorials.

### Data Extraction and Quality Assessment

A predefined standardized form was used to extract relevant information as follows: 1) demographic characteristics such as the number of patients and nodules, patient age, and male/female ratio; and 2) study characteristics such as first author, publication year, location, and institution, period of the study conducted, the mean or median size of nodules, number of readers and their experience, whether blinded to final results, reference standard, inter-reader agreement regarding ACR TI-RADS, and US features (composition, echogenicity, shape, margin, and echogenic foci). The quality assessment of included studies was performed according to the Guidelines for Reporting Reliability and Agreement Studies (GRRAS) (19), in which the following items were used to score each study: description of the title and abstract, methods, results, discussion, and auxiliary material. For individual studies, these categories were scored as high quality if it was described in sufficient detail in the article with no potential bias.

### Data Synthesis and Statistical Analysis

The summary estimates of *κ* or ICC values were calculated with the random-effects model (Sidik–Jonkman method) (20, 21) and categorized as follows: a *κ* value of <0.20 indicates slight agreement; a *κ* value between 0.21 and 0.40, fair agreement; a *κ* value between 0.41 and 0.60, moderate agreement; a *κ* value between 0.61 and 0.80, substantial agreement; and a *κ* value of between 0.81 and 1.00, almost perfect agreement. Aside from overall ACR TI-RADS classification, the *κ* values of five US features included in this guideline were pooled, namely, composition, echogenicity, shape, margin, and echogenic foci. The forest plots were created to graphically present the results. Multiple subgroup analyses regarding the following variables were performed: 1) for readers with experience of more than 10 years, 2) for readers with experience of less than 10 years or not dedicated in such area, 3) for studies providing details on nodule size ≥1 cm, and 4) for studies providing details on patients recommended for FNAB. Considering that several studies reported the head-to-head comparisons between the ACR TI-RADS with other scoring systems such as ATA guidelines and the EU TI-RADS, these guidelines were compared in available studies.

Sensitivity analyses were performed to evaluate the contribution of the individual study to the pooled estimates, by excluding each study at a time and recalculating the pooled estimates for the remaining studies. The meta-regression analysis was performed to explore the causes of heterogeneity by adding the following covariates: 1) study design (prospective vs. retrospective), 2) reference standard (FNAB vs. FNAB+thyroid resection pathology), 3) number of readers (readers = 2 vs. readers > 2), 4) number of nodules (<150 vs. ≥150), 5) malignant rate (<20% vs. ≥20%), and 6) inter-reader agreement (Cohen *κ* vs. ICC). Heterogeneity throughout studies was determined with the *Q* statistics and the inconsistency index (*I* ^2^), as follows: for values between 0% and 40%, unimportant; between 30% and 60%, moderate; between 50% and 90%, substantial; and between 75% and 100%, considerable (22). Funnel plots and the rank test were used for the assessment of any possible publication bias. All analyses were performed with STATA 16.0 (StataCorp, Texas, USA), with *p*-values <0.05 considered as statistical significance. Two reviewers (WL and YS) independently conducted the data extraction and quality assessment, and disagreements were resolved by discussion, arbitrated by a third reviewer (AD).

## Results

### Literature Search and Data Extraction

**Figure 1:**
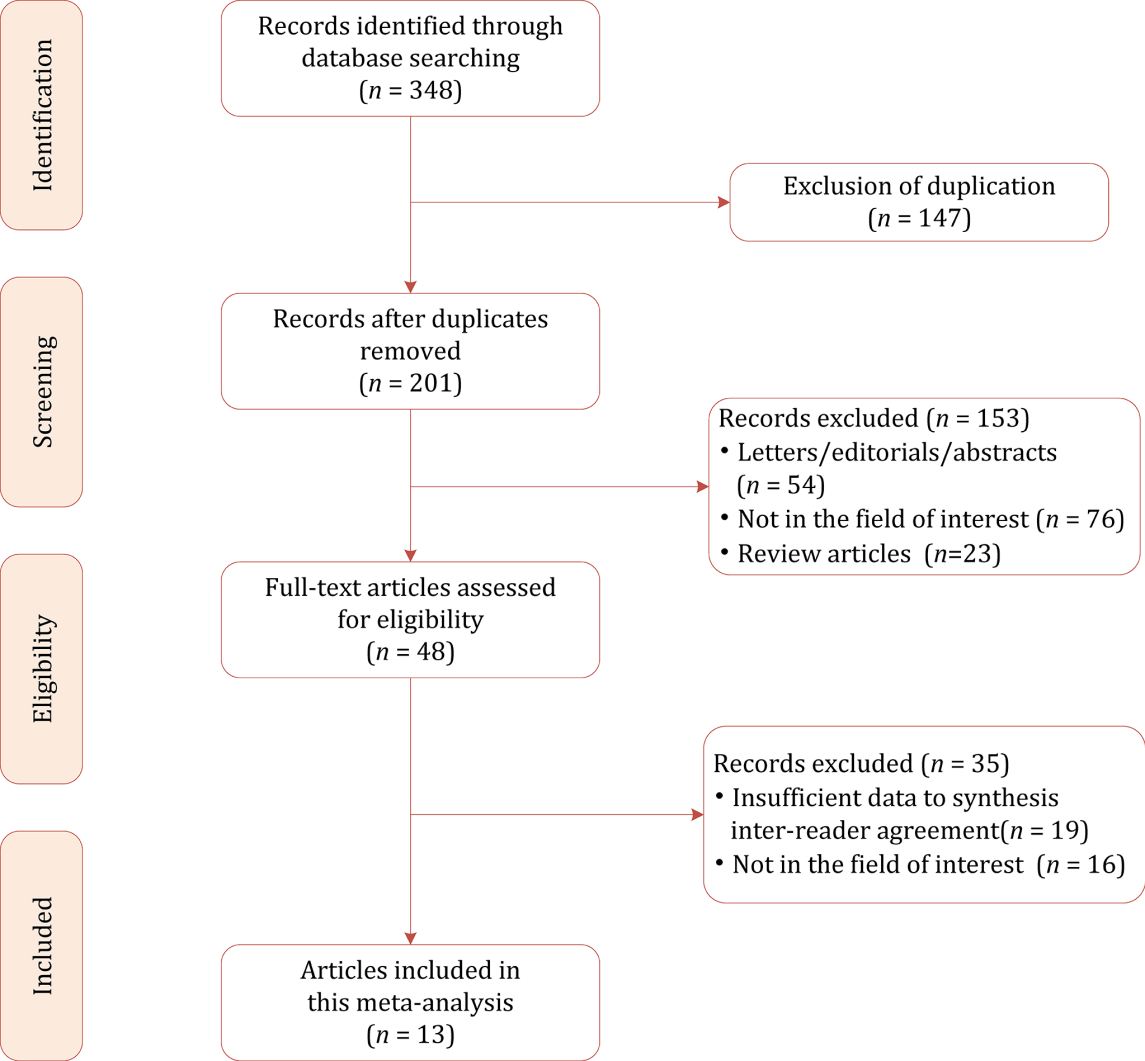
Study selection process for this systematic review and meta-analysis.

**Figure 1** shows the flowchart of the publication selection process. The literature search identified 348 references initially, of which 147 were excluded due to duplicates. After the titles and abstracts were inspected, 153 articles were excluded. The full-text review was conducted among the remaining 48 potential articles, then 19 articles were excluded due to insufficient data to synthesize inter-reader agreement, and 16 articles were excluded because they were not in the field of interest. Finally, a total of 13 articles comprising 5,238 nodules were included in the current meta-analysis and systematic review (23–35).

### Characteristics of the Included Studies

The detailed demographic and study characteristics are summarized in **Tables 1**, **2**. Concerning study design, the majority of studies were retrospective, and only two studies were prospective. The patient sample ranged from 62 to 1,947, with a nodule number of 74–1,947. In all studies, women were dominant and account for 70%–93% of the total study population. In most studies, patients were adults, whereas in one study, the participants were adolescents (30). In 3 studies, the reported statistics of the inter-reader agreement were ICCs (29, 31, 33); in 1 study, Krippendorff’s α (26); and in the remaining 9 studies, Cohen’s kappa. In four studies, the US images were interpreted by 2 radiologists (26, 30, 32, 35), whereas in the remaining 9 studies, the inter-reader agreements were produced by at least 3 radiologists. Most studies reported details on the experience level of readers, which ranged from 1 to 32 years; however, in 1 study, the exact level of readers’ experience was not provided but merely described as “experienced” (33). In addition to ACR TI-RADS, there were 3 studies that provided the inter-reader agreement of ATA guidelines and EU TI-RADS. The number of radiologists across studies ranged from 2 to 8. The mean nodule size among included studies ranged from 1.46 to 2.9 cm. Aside from overall ACR TI-RADS classification, 8 studies reported the inter-reader agreement regarding in detail five US features; moreover, 3 studies reported the inter-reader agreement recommended by the radiologists for FNAB (24, 26, 27).

**Table 1:** Demographic characteristics of included studies.

First author	Publication year	Location	Patient number	Nodule number	Malignant	M/F	Age (year, mean ± SD/IQR, range)	Size (cm, mean ± SD/IQR, range)
**Chung et al.** (24)	2020	USA	150	150	10	34/116	58 ± 15	2.0 (1.0–6.1)
**Daniels et al.** (25)	2020	USA	121	136	24	21/115	55.8 ± 14.1	2.65 ± 1.5
**Grani et al.** (26)	2018	Italy	265	501/554	NA	NA	NA	NA
**Huang et al.** (35)	2021	China	198	203	58	43/156	42.8 ± 11.2	0.8 (0.6–1.2)
**Itani et al.** (28)	2019	USA	131	180	10	31/100	51.1 ± 14.4	2.58 ± 1.21
**Lim-Dunham et al.** (30)	2019	USA	62	74	20	6/56	12.5 (9–17)/16.5 (15–18)^†^	1.90 (1.30–2.80)
**Hoang et al.** (27)	2018	USA	92	100	15	NA	52 ± 14	2.8 ± 1.3/2.2 ± 1.3
**Pandya et al.** (31)	2020	USA	1947	1947	193	475/1472	56 ± 15	1.68 ± 0.88
**Phuttharak et al.** (32)	2019	Thailand	94	108	53	7/87	51.6 ± 13.08	2.12 ± 1.2
**Seifert et al.** (34)	2019	Germany	80	80	14	24/56	50.8 ± 13.1	NA
**Basha et al.** (23)	2019	Egypt	100	948	136	66/314	45.3 ± 14.2	2.9 ± 1.37
**Li et al.** (29)	2019	China	128	130	73	27/101	47.8 ± 10.5	1.46 ± 1.0
**Sahli et al.** (33)	2019	USA	127	127	26	35/92	52 ± 14	2.3 (0.5–8.0)

IQR, interquartile range; NA, not available.

^†^ Male/female.

**Table 2:** Study characteristics of included studies.

First author	Reference standard	Study design	Period	Blinding	Reader number	Readers’ experience (year)	Agreement statistic	Agreement value
**Chung et al.** (24)	US-guided FNAB	Retrospective	Jan. 2017–Apr. 2018	Yes	6	3 readers ≥22 3 readers ≥1	Cohen κ	0.31
**Daniels et al.** (25)	FNAB+pathology	Retrospective	Jan. 2017–Aug. 2018	Yes	4	11 (5–16)	Cohen κ	0.49
**Grani et al.** (26)	US-guided FNAB	Retrospective	Jan. 2006–Jan. 2008	Yes	2	≥6	Krippendorff’s α	0.57
**Huang et al.** (35)	FNAB+pathology	Retrospective	Jan. 2015–Dec. 2018	Yes	2	17/22	Cohen κ	0.47
**Itani et al.** (28)	FNAB+pathology	Retrospective	Jan. 2014–Jun. 2014	Yes	4	5/15/20/20	Cohen κ	0.64
**Lim-Dunham et al.** (30)	US-guided FNAB	Retrospective	1996–2017	Yes	2	≥10	Cohen κ	0.29
**Hoang et al.** (27)	FNAB+pathology	Retrospective	Apr. 2009–May 2010	Yes	8	3–32	Cohen κ	0.38
**Pandya et al.** (31)	US-guided FNAB	Retrospective	Oct. 2009–Feb. 2016	Yes	5	4–32	ICC	0.86
**Phuttharak et al.** (32)	US-guided FNAB	Retrospective	Jan. 2015–Oct. 2017	Yes	2	1 reader ≥2	Cohen κ	0.71
						1 reader ≥10		
**Seifert et al.** (34)	US-guided FNAB	Prospective	NA	Yes	4	1 reader ≥3	Cohen κ	0.56
						3 readers ≥5		
**Basha et al.** (23)	US-guided FNAB	Prospective	May 2017–Dec. 2018	Yes	3	≥15	Cohen κ	0.44
**Li et al.** (29)	FNAB+pathology	Retrospective	Dec. 2016–Mar. 2018	Yes	4	≥5	ICC	0.51
**Sahli et al.** (33)	US-guided FNAB	Retrospective	Feb. 2012–Sep. 2016	Yes	3	Experienced	ICC	0.57

FNAB, fine-needle aspiration biopsy; ICC, intraclass correlation coefficient; NA, not available; US, ultrasound.

### Quality Assessment

All included studies were scored as high quality according to the criteria evaluated. The majority of studies reported that they included the consecutive patient population, however, which was not reported explicitly in one study (34). In one study, the readers’ experience levels were unavailable (33). Further details on the study quality are provided in **Table S1**.

### Pooled Inter-Reader Agreement of American College of Radiology Thyroid Imaging Reporting and Data System

**Figure 2:**
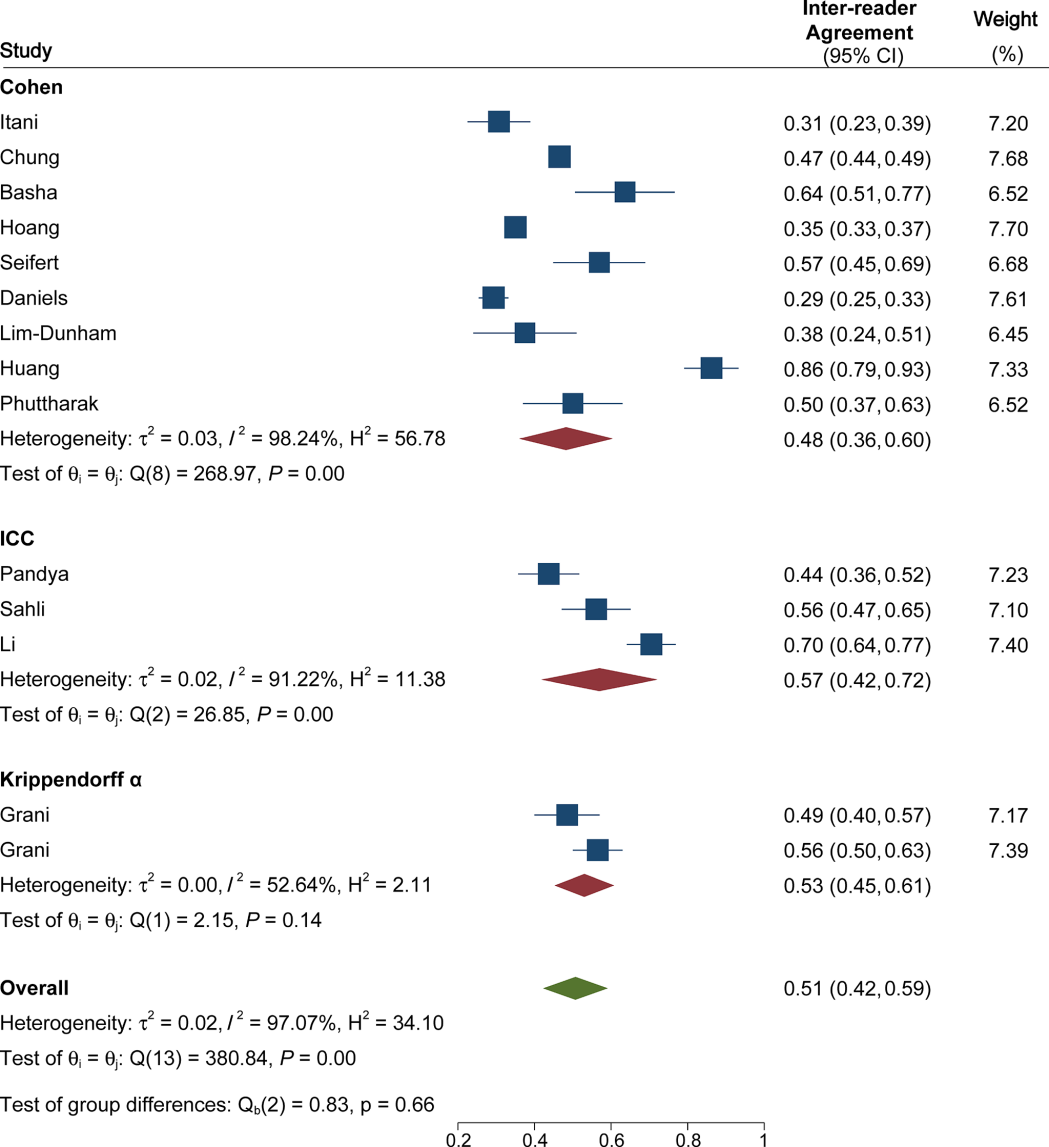
Coupled forest plot of pooled inter-reader agreement of the ACR TI-RADS for classification of thyroid nodules. ACR, American College of Radiology; TI-RADS, Thyroid Imaging Reporting and Data System.

**Figure 3:**
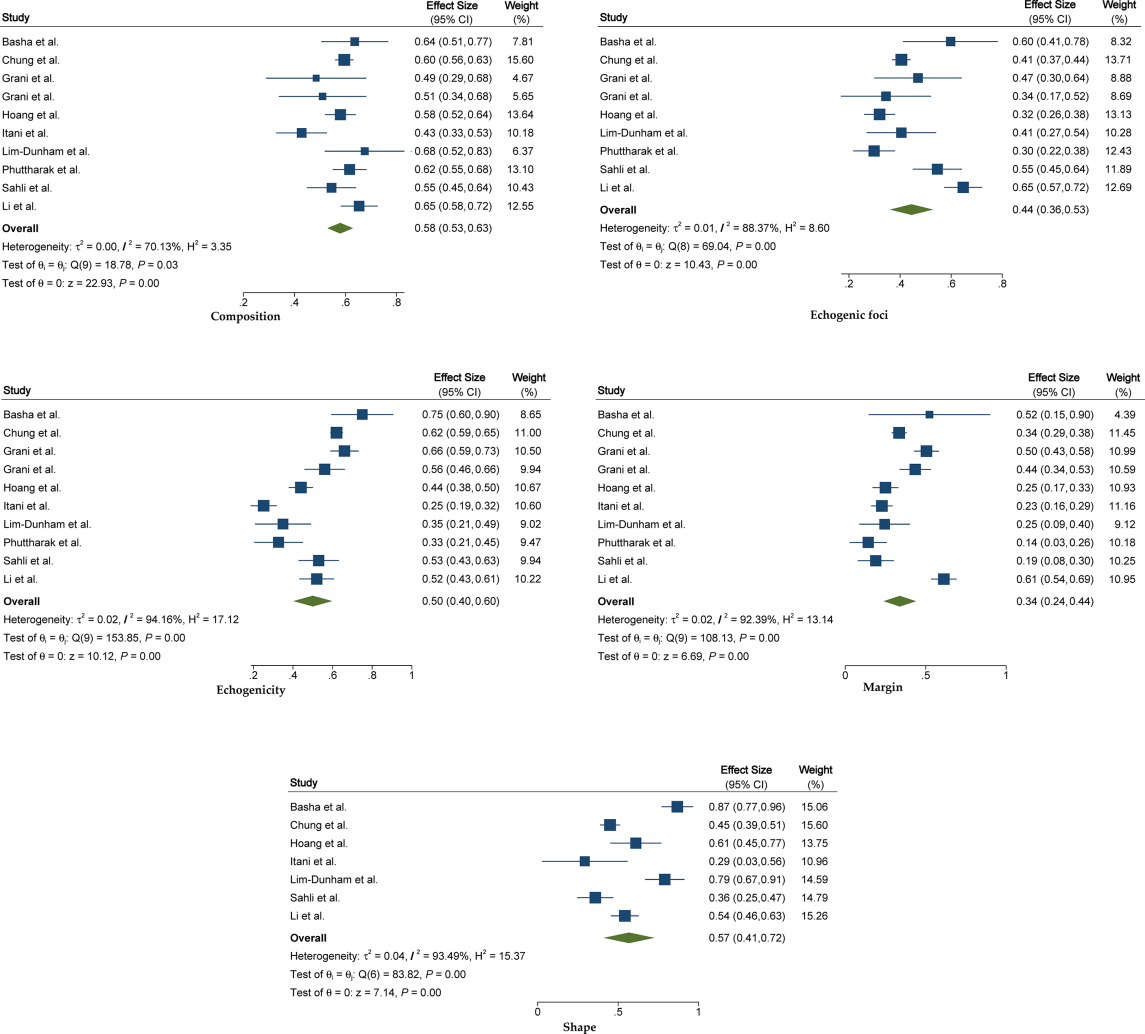
The forest plot of pooled inter-reader agreement for five US features recommended by the ACR TI-RADS. US, ultrasound; ACR, American College of Radiology; TI-RADS, Thyroid Imaging Reporting and Data System.

**Figure 4:**
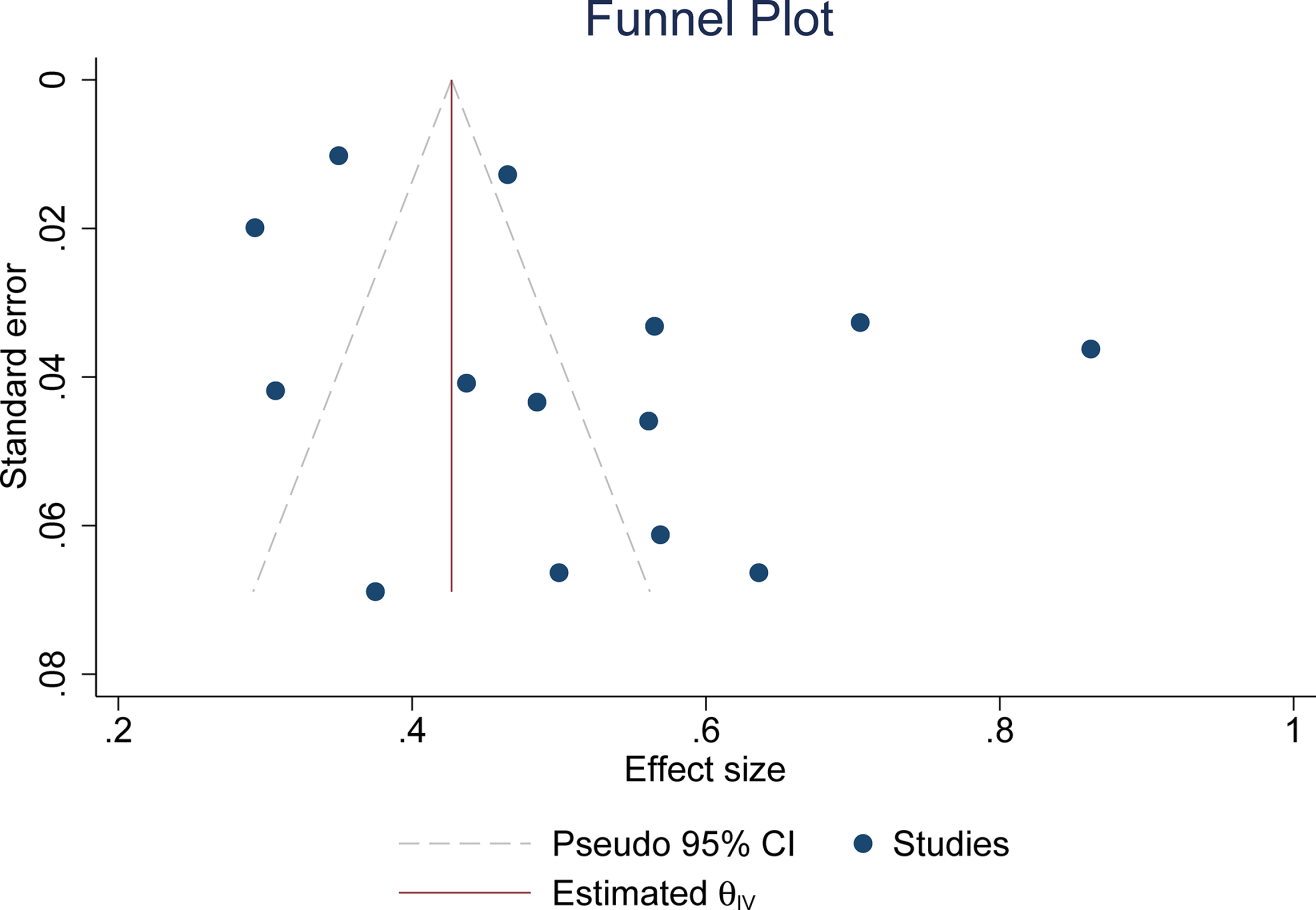
The funnel plot for testing publication bias.

The pooled summary estimates of inter-reader agreement of the ACR TI-RADS are summarized in **Figure 2**. For individual studies, the *κ* values ranged from 0.29 to 0.86, and the pooled summary estimates of the *κ* value were 0.51 (95% CI 0.42–0.59) for the overall ACR TI-RADS classification. For 5 major US features, the highest inter-reader agreement was composition, with pooled *κ* of 0.58 (95% CI 0.53–0.63), followed by shape (*κ* = 0.57, 95% CI 0.41–0.72). The poorest inter-reader agreement was margin (*κ* = 0.34, 95% CI 0.24–0.44). For the other 2 features, echogenicity and echogenic foci, the pooled *κ* values were 0.50 (95% CI 0.40–0.60) and 0.44 (95% CI 0.36–0.53), respectively (**Figure 3**). We did not find substantial difference between the ACR TI-RADS with EU TI-RADS (0.54, 95% CI 0.49–0.58 vs. 0.54, 95% CI 0.32–0.77, *p* = 0.95) and ATA guidelines (0.51, 95% CI 0.45–0.58 vs. 0.54, 95% CI 0.45–0.64, *p* = 0.60). There was no significant publication bias regarding the ACR TI-RADS classification among included studies (*p* = 0.47, **Figure 4**). Likewise, there was no significant publication bias regarding the five US features, with *p*-values of 0.43–0.97.

### Subgroup Analyses and Meta-Regression Analysis

For experienced readers, the pooled *κ* value based on 3 studies was 0.46 (95% CI 0.30–0.62), whereas for inexperienced readers, the inter-reader agreement was higher, with pooled *κ* value of 0.54 (95% CI 0.32–0.76). However, the difference did not reach statistical significance. For 3 studies reporting inter-reader agreement for thyroid nodules ≥1 cm, the pooled *κ* value of 0.53 (95% CI 0.44–0.63) was suggested to be higher than the overall *κ* value. The pooled summary estimates of inter-reader agreement in sensitivity analysis were similar to those of all studies, with substantial heterogeneity across included studies (**Table S2**). Meta-regression analysis was performed to investigate the cause of heterogeneity; among the various potential factors, we found that only the malignant rate (<0.2 vs. ≥0.2) was significantly associated with the heterogeneity (*p* = 0.03). All of the other covariates were not substantial factors, with *p*-values ranging from 0.08 to 0.96; the details are presented in **Table 3**.

**Table 3:** Meta-regression analysis.

Item	Value	Pooled inter-reader agreement	*p*
Study design	Prospective	0.60 (95% CI 0.51–0.69)	0.08
	Retrospective	0.49 (95% CI 0.41–0.58)	
Size	≥1	0.54 (95% CI 0.37–0.70)	0.71
	All	0.50 (95% CI 0.40–0.60)	
Reference	FNAB	0.51 (95% CI 0.46–0.55)	0.96
	FNAB+pathology	0.50 (95% CI 0.32–0.68)	
Readers	=2	0.56 (95% CI 0.39–0.73)	0.36
	>2	0.47 (95% CI 0.39–0.55)	
Patient number	<150	0.46 (95% CI 0.36–0.56)	0.15
	≥150	0.57 (95% CI 0.45–0.70)	
Malignant rate	<0.2	0.42 (95% CI 0.35–0.49)	0.03
	>0.2	0.61 (95% CI 0.45–0.76)	
Nodule number	≤150	0.47 (95% CI 0.39–0.56)	0.43
	>150	0.55 (95% CI 0.39–0.71)	
Scoring system	ACR vs. EU	0.54 (95% CI 0.49–0.58)	0.95
		0.54 (95% CI 0.32–0.77)	
	ACR vs. ATA	0.51 (95% CI 0.45–0.58)	0.60
		0.54 (95% CI 0.45–0.64)	
Agreement statistic	Cohen	0.49 (95% CI 0.41–0.57)	0.39
	ICC	0.57 (95% CI 0.41–0.73)	

ACR, American College of Radiology; ATA, American Thyroid Association guidelines; EU, European Union; FNAB, fine-needle aspiration biopsy; ICC, intraclass correlation coefficient; TI-RADS, Thyroid Imaging Reporting and Data System.

## Discussion

In this study, we systematically assessed the inter-reader agreement for the ACR TI-RADS for the classification of thyroid nodules. Based on 13 studies, the pooled *κ* value of 0.51 (95% CI 0.42–0.59) revealed that the ACR TI-RADS has moderate inter-reader agreement among the varied experience of radiologists. Aside from overall ACR TI-RADS classification, most studies reported the inter-reader agreement regarding the detailed five US features recommended by the guideline. Of them, the highest was composition (*κ* = 0.58), whereas the lowest was margin (*κ* = 0.34). For other features, the pooled *κ* values ranging from 0.44 to 0.57 indicated moderate inter-reader agreement. In light of several studies that provided a head-to-head comparison between ACR TI-RADS and EU TI-RADS as well as ATA guidelines, we made a direct comparison in available studies, and the results suggested that there was no substantial difference between these 3 guidelines (*p* = 0.95 and *p* = 0.60, respectively). However, both comparisons were based on 3 studies; therefore, the results should be interpreted with caution. In a previous meta-analysis by Liu et al., which included 7 studies, the pooled inter-reader agreement was slightly higher, with 0.54 (95% CI 0.49−0.5). However, this only provided an indirect comparison; moreover, only 3 studies included in their meta-analysis involved the ACR TI-RADS (36).

As substantial heterogeneity throughout studies was observed, we performed meta-regression analysis to look into the sources. Among the various factors, we found that only the malignant rate (<0.2 vs. ≥0.2) was significantly associated with heterogeneity (*p* = 0.03), whereas all other covariates were not significant factors that contributed to the heterogeneity. Nevertheless, we found a substantial difference in inter-reader agreement regarding study design (0.60 for prospective vs. 0.49 for retrospective), even though it was not found to be statistically significant (*p* = 0.08). In the current study, multiple subgroup analyses were performed to account for several clinical settings. We noted that there was no significant difference between experienced readers and inexperienced readers (*p* = 0.55); moreover, our analysis showed that the inter-reader agreement in inexperienced readers was even higher than in experienced readers (0.53 vs. 0.45). One explanation may be that those with less experience were more likely to strictly use the ACR TI-RADS, while those observers with more experience would be influenced by their prior experience (24). In the study by Daniels et al., they observed that most experienced readers had the lowest intra-reader agreement (25). To address this problem, Seifert et al. and Grani et al. conducted training and demonstrated that the reproducibility and inter-reader agreement were improved on overall ACR TI-RADS classification (24, 26).

Since Kwak et al. proposed the first TI-RADS in 2005 (12), several versions of scoring systems based on this guideline were developed, including the EU, ACR, and Korean TI-RADS (14, 16, 37). Different from other classification systems based on patterns, the ACR TI-RADS is a point-based risk stratification system in which nodules are scored according to five US features. According to this guideline, the more features that a nodule possesses, the more points will accumulate, and the total points determine the nodule’s final ACR TI-RADS classification. We performed direct comparison in studies providing a head-to-head comparison between ACR TI-RADS with ATA guidelines and EU TI-RADS, and the results demonstrated that there was no significant difference among these guidelines even though these comparisons were based on a few studies. Aside from inter-reader agreement, previous studies revealed no significant difference in diagnostic performance between these guidelines, irrespective of sensitivity and specificity (38).

In view of most included studies reporting details on US features of composition, echogenicity, shape, margins, and echogenic foci that are recommended by the ACR TI-RADS, we thus investigated the inter-reader agreement regarding these US features. According to our analysis, the poorest inter-reader agreements were margin and echogenic foci, which had only fair (*κ* = 0.34) and moderate (*κ* = 0.44) reproducibility, respectively. One possible explanation was that a suspicious margin or echogenic foci may have been apparent on only a few of the static images or only could be observed in the video clip. By comparison, composition and echogenicity had higher reproducibility, as findings present throughout the nodule and are likely to be seen on more images, according to our analysis, which had a nearly substantial agreement, with pooled *κ* values of 0.57 and 0.58.

### Limitations

There are some limitations to our study. First, most included studies were retrospective in study design, leading to a high risk of bias for the patient selection domain. Nevertheless, because of insufficient data, it is unfeasible to pool the summary estimates from merely two prospective studies. Second, substantial heterogeneity existed among included studies, which affected the general applicability of this systematic review. We performed multiple subgroup analyses and meta-regression, and the results indicated that the malignancy rate was the only significant factor that contributed to the heterogeneity. However, these analyses only explained part of the heterogeneity, and these analyses were based on only a few studies; thus, the results should be interpreted cautiously.

## Conclusion

The ACR TI-RADS demonstrated moderate inter-reader agreement between radiologists for the overall classification. However, the US feature of margin recommended by this guideline only showed fair inter-reader reliability among different observers.

## Data Availability Statement

The original contributions presented in the study are included in the article/**Supplementary Material**. Further inquiries can be directed to the corresponding author.

## Author Contributions

Guarantor of the article: AD. Conception and design: WL and YS. Collection and assembly of data: HX, WS. Data analysis and interpretation: WL and HX. All authors contributed to the article and approved the submitted version.

## Conflict of Interest

The authors declare that the research was conducted in the absence of any commercial or financial relationships that could be construed as a potential conflict of interest.

## Publisher’s Note

All claims expressed in this article are solely those of the authors and do not necessarily represent those of their affiliated organizations, or those of the publisher, the editors and the reviewers. Any product that may be evaluated in this article, or claim that may be made by its manufacturer, is not guaranteed or endorsed by the publisher.
