# Supplementary material for: Systematic Review and Meta-Analysis of American College of Radiology TI-RADS Inter-Reader Reliability for Risk Stratification of Thyroid Nodules
Source: Front Oncol. 2022 May 13;12:840516. doi: 10.3389/fonc.2022.840516 (PMC9136001; doi:10.3389/fonc.2022.840516)
Supplement: Supplementary file 1 [file Table_1.docx]

**Table S1.** Summary of the Quality of the Included Studies

| **Study** | **Item 1** | **Item 2** | **Item 3** | **Item 4** | **Item 5** | **Item 6** | **Item 7** | **Item 8** | **Item 9** | **Item 10** | **Item 11** | **Item 12** | **Item 13** |
| --- | --- | --- | --- | --- | --- | --- | --- | --- | --- | --- | --- | --- | --- |
| **Chung et al.** | high | high | high | high | high | low | high | high | high | high | high | high | high |
| **Daniels et al.** | high | high | high | high | high | high | high | high | high | high | high | high | high |
| **Grani et al.** | high | high | high | high | high | high | high | high | high | high | high | low | high |
| **Huang et al.** | high | high | high | high | high | high | high | high | high | high | high | high | high |
| **Itani et al.** | high | high | high | high | high | high | high | high | high | high | high | high | high |
| **Lim-Dunham et al.** | high | high | high | high | high | high | high | high | high | high | high | high | high |
| **Hoang et al.** | high | high | high | high | high | high | high | high | high | high | high | unclear | high |
| **Pandya et al.** | high | high | high | high | high | high | high | high | high | high | high | high | high |
| **Phuttharak et al.** | high | high | high | high | high | high | high | high | high | high | high | high | high |
| **Seifert et al.** | high | high | high | high | high | unclear | high | high | high | high | high | unclear | high |
| **Basha et al.** | high | high | high | high | high | low | high | high | high | high | high | high | high |
| **Li et al.** | high | high | high | high | high | high | high | high | high | high | high | high | high |
| **Sahli et al.** | high | high | high | high | high | high | high | high | high | high | high | unclear | high |

**Item 1**: Identify in title or abstract that inter-rater/intra-rater reliability or agreement was investigated;

**Item 2**: Name and describe the diagnostic or measurement device of interest explicitly;

**Item 3**: Specify the subject population of interest;

**Item 4**: Specify the rater population of interest (if applicable);

**Item 5**: Describe what is already known about reliability and agreement and provide a rationale for the study (if applicable);

**Item 6**: Explain how the sample size was chosen. State the determined number of raters, subjects/objects, and replicate observations;

**Item 7**: Describe the sampling method;

**Item 8**: Describe the measurement/rating process;

**Item 9**: State whether measurements/ratings were conducted independently;

**Item 10**: Describe the statistical analysis;

**Item 11**: State the actual number of raters and subjects/objects that were included and the number of replicate observations that were conducted;

**Item 12**: Describe the sample characteristics of raters and subjects;

**Item 13**: Report estimates of reliability and agreement including measures of statistical uncertainty
