# Supplementary material for: Systematic Review and Meta-Analysis of American College of Radiology TI-RADS Inter-Reader Reliability for Risk Stratification of Thyroid Nodules
Source: Front Oncol. 2022 May 13;12:840516. doi: 10.3389/fonc.2022.840516 (PMC9136001; doi:10.3389/fonc.2022.840516)
Supplement: Supplementary file 2 [file Table_2.docx]

**Table S2.** Sensitivity Analysis

| **Study** | **Size (95% CI)** | ***I*^2^** |
| --- | --- | --- |
| ***All studies*** | **0.51/0.42-0.59** | **96.6** |
| Excluding |  |  |
| Itani | 0.52/0.44-0.60 | 96.8 |
| Grani (Set 501) | 0.51/0.43-0.59 | 96.8 |
| Grani (Set 554) | 0.50/0.42-0.58 | 96.7 |
| Chung | 0.51/0.41-0.61 | 96.7 |
| Basha | 0.50/0.42-0.58 | 96.8 |
| Daniels | 0.52/0.44-0.61 | 96.4 |
| Lim-Dunham | 0.52/0.43-0.60 | 96.8 |
| Huang | 0.48/0.41-0.54 | 94.8 |
| Li | 0.49/0.42-0.57 | 96.1 |
| Sahli | 0.50/0.42-0.58 | 96.8 |
| Pandya | 0.51/0.43-0.60 | 96.8 |
| Hoang | 0.52/0.43-0.61 | 95.8 |
| Seifert | 0.50/0.42-0.58 | 96.8 |
| Phuttharak | 0.51/0.43-0.59 | 96.8 |
